# Exploring the mediating role of PsyCap in the relationship between orientation training and work engagement: the perspective of COR and SRT theories

**DOI:** 10.3389/fpsyg.2023.1263658

**Published:** 2023-09-25

**Authors:** Zibin Song, Houchen Zhang, Jie Li

**Affiliations:** School of Tourism, Hainan University, Haikou, Hainan, China

**Keywords:** conservation of resources (COR), socialization resources theory (SRT), orientation training, work engagement, psychological capital (PsyCap), leader-member exchange (LMX)

## Abstract

In the domain of organizational socialization, a new research paradigm and trend concerns work engagement path way to studying newcomer assimilation through the lens of *socialization resource theory* (SRT). Drawing on theories of SRT and COR (*conservation of resources*), the present study develops and validates an integrative model, in which the effect of orientation training on work engagement involves the mediator of PsyCap and moderators of leader-member exchange (LMX) and education. SmartPLS 3.0 was used to analyze the data with 567 respondents with 5,000 bootstraps from 3- to 5-star hotels in Mainland China. The empirical results indicate that newcomers’ PsyCap fully mediates the relationship between orientation training and work engagement. They also suggest that education and LMX, respectively, moderate the effects of orientation training on PsyCap and work engagement. Theoretical and practical implications of these findings are drawn in the context of organizational socialization and human resources development.

## 1. Introduction

Work engagement is a positive work state characterized by energy, focus, and dedication (Bakker and Sanz-vergel, 2014). It plays a critical role in both enhancing sustainable competitiveness and profitability in businesses (Saks, 2017) and achieving successful outcomes of newcomers’ organizational socialization (Saks and Gruman, 2018). In the hospitality industry, employee engagement is particularly important (Hassanein and Özgit, 2022; Liu et al., 2023) for one notable reason. That is, hotel organizations are labor-intensive and service-oriented, and they must cultivate high levels of work engagement among staff to meet customers’ needs (Asghar et al., 2021). Saks (2017) reports that, however, there is an overall declining trend in employee engagement globally. For example, only 24% of workers in Southeast Asia, as per a report^1^, are seen to be engaged at work. Evidence shows that poor engagement is linked to low job satisfaction and can even lead to employees’ turnover (Asghar et al., 2021; Ozturk et al., 2021). As such, it is necessary for managers to fully understand and intervene in the proximal and distal factors that lead to the levels of work engagement.

According to Boswell et al. (2005), newcomers’ engagement levels often decline to varying degrees after entering an organization in organizational socialization. This phenomenon is known as the “hangover effects” (Saks and Gruman, 2018). Most newcomers experience the hangover effects unless organizational managers proactively adopt effective socialization practices to develop and maintain newcomers’ higher levels of work engagement during the socialization process (Saks and Gruman, 2018; Liu et al., 2023). In other words, the socialization practices largely determine whether newcomers’ level of work engagement decreases, stabilizes, or increases (Saks and Gruman, 2018). Moreover, maintaining and developing newcomers’ work engagement is considered as an effective management approach to achieving success-related outcomes of organizational socialization (Saks and Gruman, 2018; Liu et al., 2023). According to Ashforth et al. (2007), organizational socialization could be referred to as the process by which a newcomer transforms from a rank outsider into an effective insider. It significantly affects newcomers’ careers (Saks and Gruman, 2011) and spurs the company’s profit growth (Strack et al., 2012). As a result, for more than three decades, both partitioners and scholars have interests in issues relevant to newcomers’ organizational socialization (Peltokorpi et al., 2022).

Saks and Gruman (2018) have theoretically developed a model of socialization resources theory, focusing on the work engagement pathway to newcomers’ organizational socialization. Their model includes both proximal (e.g., PsyCap, Saks and Gruman, 2011, 2018) and distal (e.g., supervisor support, Saks and Gruman, 2018) factors that lead to newcomers’ work engagement. SRT theory argues that newcomers should be provided with the resources they need to integrate into their employment organization effectively. In fact, the engagement path way to socialization is a new paradigm for both socialization research and practice. As a new approach, SRT is seen to have its limitations. Existing SRT models have, for example, neglected potential moderating variables such as mentoring (Cai et al., 2021) and income (Liu et al., 2023). Furthermore, there has been a lack of empirical research on resource caravans and resource passageways. Resource caravans refer to the interconnection and influence of organizational and individual resources as a moving “caravan,” while resource passageways are specific environments in which resources are nourished or hindered by the width or narrowness of the “passageway” (Hobfoll, 2011; Hobfoll et al., 2018). In this regard, although SRT theory points out the important role of resource caravans and resource passageways in the process of organizational socialization, most of the current empirical studies based on SRT theory focus on individual socialization resources rather than socialization resource caravans, albeit a few studies (e.g., Liu et al., 2023) have explored the role of resource passageways (e.g., leader member-exchange, LMX) as well as their influences on newcomer socialization outcomes.

Orientation training refers to institutionalized training program tailored to newcomers right after their entry into the employment organization (Saks and Gruman, 2011). The effectiveness of orientation training is vital for both newcomers and their organizations (Zheng, 2018). It is regarded as an indispensable component in achieving success-related socialization outcomes in organizational context (Saks and Gruman, 2018; Raub et al., 2021). Klein and Weaver (2000) note that, for example, newcomers with orientation experiences assimilate better into the organization than their counterparts without orientation experiences do. A valid orientation training program enables new hires to become familiar quickly with the job’s numerous facets, including organizational practices, culture, and task duties, among others (Song and Chathoth, 2010). In turn, the familiarity enables newcomers to quickly adjust to their job positions (Tabvuma et al., 2015). In addition, effective orientation training acts as a “rope” to ease newcomers’ transition from “outsider” to “insider” and help them deal with a variety of stressors (Saks and Gruman, 2011). Although previous studies have explored the relationship between training and work engagement, scholars have not yet come to a consistent and clear conclusion. For instance, Shantz et al. (2016) note that work engagement in the sample of health care can be predicted by training, while Almotawa and Shaari (2020) report that the same causality is not statistically significant. This would suggest that effect of orientation training on work engagement could be indirect, but not direct, albeit in SRT model (Saks and Gruman, 2018) socialization resources (e.g., orientation training, Saks and Gruman, 2011) are proposed to have both direct and indirect effects on work engagement. This would suggest that more empirical evidence should be provided to tell how and why orientation training affects work engagement.

According to SRT, organizational job resources often influence work engagement in two ways (Saks and Gruman, 2018). The first is that socialization resources have direct influences on work engagement (Liu et al., 2023). The second is that socialization resources indirectly affect work engagement through PsyCap. According to Luthans and Youssef-Morgan (2017) and Luthans et al. (2007), PsyCap is an individual’s positive development state, which could be understood as a cluster/caravan formed by four HERO (Hope, Efficacy, Resilience, and Optimism) resources: (1) perseverance toward goals and, when necessary, making certain adjustments to achieve success (Hope), (2) the confidence to undertake and exert the necessary effort to complete challenging tasks (Efficacy), (3) when plagued by problems and adversity, the ability to stay motivated and even rise above oneself to succeed (Resilience), and (4) positive attributions about present and future success. Prior research demonstrates that people’s PsyCap is effective in reducing job stress (Patnaik et al., 2022), and it mediates the relationship between stress and job satisfaction (Xie et al., 2021). In addition, PsyCap mediates the effect of organizational trust on job performance (Zhang et al., 2021). To our knowledge, there has been no documented report on PsyCap’s mediating role in the relationship between orientation training and newcomers’ work engagement. As per COR theory, organizational and individual resources may cross over in the resource corridor and produce an impact known as the crossover effect (Hobfoll et al., 2018). Therefore, it is likely that PsyCap is influenced by orientation training and further impacts work engagement. It should be noted that PsyCap in our study is considered a factor consisting of the forgoing four specific dimensions. This is different from some previous empirical studies (e.g., Prayag et al., 2020) that the first-order four dimensions of PsyCap are investigated only. In other words, our study shifts the focus on PsyCap from the individual resource perspective to the cluster of resource perspective. Our doing is in line with COR theory, and thus it is likely to better capture the true dynamic of newcomers’ PsyCap (Vilariño del Castillo and Lopez-zafra, 2022).

Additionally, in response to the lack of existing moderating variables in organizational socialization (Cai et al., 2021), our study explores the moderating effects of leader-member exchange (LMX) and education. LMX represents the quality of the relationship between leaders and subordinates (Graen and Uhl-Bien, 1995) and is also critical in influencing the attitudes and behaviors of both parties (Xu and Li, 2019). Due to the differentiated nature of the leader-subordinate relationship, previous research has shown that LMX can impact employees’ work engagement. For example, Ke and Ding (2020) found that LMX moderated the effect of entrepreneurial leaders on work engagement. In addition, LMX can be viewed as a resource caravan passageway and may influence the flow of resources within the gallery. Therefore, newcomers in a high-quality LMX context will more effectively use orientation training as a resource to maintain work engagement. Besides, education refers to the level of education received by an individual. It meets the criteria for categorizing resources under the COR theory, and the information it provides can assist people in acquiring other resources they may need (Hobfoll, 1989). In short, our study is based on COR theory, and it proposes an integrative model of organizational socialization by which PsyCap mediates the relationship between orientation training and work engagement. Meanwhile, some direct effects in our model are likely to be moderated by LMX and newcomers’ education levels. Specifically, our research is focused on the following three research questions:

1. Does orientation training impact newcomers’ work engagement through PsyCap?
2. Does LMX moderate the relationship between orientation training and work engagement?
3. Does a newcomer’s education moderate the relationship between orientation training and PsyCap?

The present study will substantially contribute to the literature in two ways. First, exploring the mediating role of PsyCap in the relationships between orientation training and work engagement enables the present study to tell how and why organizational resources affect newcomers’ work engagement. Second, exploring the potential moderation roles of LMX and education enables the present study to understand under what conditions orientation training affects work engagement and PsyCap, respectively.

## 2. Literature review and research hypothesis

### 2.1. The COR theory and research framework

Initially, COR theory seeks to provide a new research perspective in understanding “what stress is” (Hobfoll, 1989, 2011). It contends that individuals tend to conserve, protect, and acquire resources, and on this basis, both potential and actual resource losses can cause stress at the individual level (Hobfoll, 1989; Hobfoll et al., 2018). In comparison to veteran employees, newcomers usually have few opportunities to gain resources because of their relatively short tenure in the new organization. Moreover, new employee groups have limited resource reserves, and integration into the new company requires a large amount of personal and organizational resources. More often than not, newcomers are frequently seen to suffer from resource loss and a relative resource imbalance. The resource gain paradox states that in the face of resource loss, timely resource replenishment is more crucial and valuable to individuals (Hobfoll et al., 2018). At this point, replenishing those already low on resources with fresh ones can help them promptly and do more to stop the ongoing loss of resources (Hobfoll et al., 2018). For individuals with scarce resources, the replenishment and supply of resources are helpful for relieving stress and tension.

First, from the perspective of COR (Hobfoll et al., 2018), job resources influence work engagement in a motivating way. A valid orientation program is an important form of job resources, and it enables newcomers to accumulate needful resources following their entry into the employment organization. This initial gain is favorable to developing resource gain spirals (Hobfoll et al., 2018), which means that a person’s initial resource accumulation enhances later resource gain (Hobfoll et al., 2018). In other words, employees with more initial individual resources will have an advantage of greater resource gain.

Second, according to the crossover effect of resources (Hobfoll et al., 2018), the impact of orientation training on work engagement is likely to be realized through personal resources (PsyCap). In this respect, SRT theory (Saks and Gruman, 2018) also notes that socialization resources affect personal resources, which in turn have an influence on newcomers’ distal socialization outcomes including work engagement organizational commitment, and turnover intention, among others. As a result, this work incorporates newcomers’ PsyCap as a particular mediator in the model, which will aid in elucidating the principles governing resource cluster operation.

Last but not least, it is also clear how LMX and education level fit into the job engagement paradigm. The leader-member exchange theory (Graen and Uhl-Bien, 1995) postulates that “in-groups” have a greater quality of leadership member exchange than “out-groups”; and that, as a result, employees may receive more support in the form of psychological or material resources. While low-quality LMX is likely to obstruct the access to the flow of resources and negatively impact engagement, high-quality LMX acts as wide resource caravan routes to facilitate the smooth passage of resource caravans (Hobfoll et al., 2018; Liu et al., 2023). Furthermore, education can be seen as an energy resource that serves to help newcomers acquire more resources (Hobfoll, 1989) and preserve and enhance personal resource pools, so it is likely to be a vital moderator at the individual level.

### 2.2. Orientation training and PsyCap

According to Luthans et al. (2007), PsyCap is highly malleable and inclusive and, therefore, can be consciously cultivated through certain specific measures. On this basis, orientation training is one of the essential methods for developing PsyCap (Saks and Gruman, 2011). In contrast, from the existing literature, there still needs to be more evidence on the relationship between orientation training and PsyCap. However, the causal relationship between orientation training and the lower-level components of PsyCap has made some progress. For example, Ashforth et al. (2012) argue that orientation training keeps newcomers optimistic and hopeful by enhancing their job competencies. Moreover, a recent investigation by Huang and Hung (2022) in high-tech companies notes that orientation training provided by organizations has a positive impact on developing employees’ motivation and self-efficacy and helps to improve their cognitive level and job performance. This is because newcomers can quickly grow accustomed to the workplace and become competent in their job duties through orientation training. Such positive experiences, in turn, give newcomers courage to face and overcome difficulties in career development. Based on the foregoing, it is reasonable to predict that orientation training is likely to positively impact PsyCap as well. This is because PsyCap is the caravan resource of the four dimensions including self-efficacy, resilience, hope, and optimism (Luthans et al., 2008). Based on the foregoing, the first hypothesis of our study is developed:

*Hypothesis 1*. Orientation training significantly and positively affects newcomers’ PsyCap.

### 2.3. PsyCap and work engagement

The COR theory states that individuals who possess sufficient resources can better manage stress and have a greater desire for work productivity (Hobfoll, 1989). Individuals will also attempt to nurture resource gain spirals to enhance their resource reserves (Wang et al., 2019). As one’s positive psychological resources, PsyCap may provide employees the inner strength and security required to work (Li et al., 2015). For instance, employees with high PsyCap have greater confidence in their talents and abilities (Zhou et al., 2019). They also maintain optimism about the present and the future and are less likely to become trapped in challenging circumstances, demonstrating higher work engagement (Zhou et al., 2019). In addition to the objective benefits that psychological capital brings to employees mentioned above, employees with high PsyCap are more willing to devote themselves to work that brings resource returns to accelerate the resource gain spiral, thus showing high dedication. Therefore, it is reasonable to speculate in our study that the higher the PsyCap of newcomers, the more engaged they will be at work. Accordingly, our study proposes the second hypothesis:

*Hypothesis 2*. PsyCap has a positive effect on newcomers’ work engagement.

### 2.4. The mediating role of PsyCap

In the literature, PsyCap is often considered as a mediator between resources and outcomes in the organizational context. Ngo et al. (2023), for example, investigate the mediating role of PsyCap in the relationship between employees’ development policies and their reported well-being. In the context of organizational socialization, newcomers might use orientation training to foster their PsyCap (noted earlier) on the one hand; and on the other hand, individuals with high PsyCap may invest in resources and show high engagement at work to increase the possibility of obtaining potential future resources (e.g., organizational recognition, income, and job promotions). Jointly, these would suggest the mediating role of PsyCap in the process of developing and maintaining newcomers’ work engagement. This concurs with Luthans et al.’s (2007) theoretical notion that PsyCap is often treated as a mediator in the relationships between job resources and outcomes. This also concurs with Saks and Gruman’s (2018) argument that socialization resources nourish newcomers’ PsyCap and further maintain and develop work engagement. A review of the literature indicates that there has been a lack of empirical evidence on the mediating role of PsyCap in the foregoing relationships. We, therefore, propose the third hypothesis:

*Hypothesis 3*. PsyCap plays a substantial mediating role between orientation training and work engagement.

### 2.5. The moderating role of LMX and education

According to COR theory, resources are constantly present in the environment, climate, and leadership that are unique to the organization. These factors can either support and nurture the resources in the “passageway” or obstruct and restrict them (Hobfoll et al., 2018). Among them, LMX is considered a resource caravan passageway (Hobfoll et al., 2018). Specifically, employees who maintain high levels of exchange with their leaders are frequently perceived as “insiders,” with interactions characterized by higher levels of support, trust, and respect. In contrast, employees who exhibit low exchange relationships tend to be viewed as “outsiders,” and their interactions are only within the formal work context (Ke and Ding, 2020).

Therefore, our study hypothesizes that newcomers with high quality LMX are more likely to access and utilize orientation training resources and thus exhibit higher levels of work engagement than those with low-quality LMX. In a high-quality LMX, leaders provide tangible and intangible job support to their subordinates, making employees feel more trusting and secure (Ke and Ding, 2020). It is crucial for organizations to enable their new associates to feel comfortable with the orientation training, which in turn promotes higher level of work engagement. Besides helping newcomers grasp the training material, high-quality LMX can give the employees more positive feedback on their work. This in turn helps motivate newcomers to exhibit high level of work engagement. In the literature, LMX is usually considered as a moderator. Xu et al. (2022), for instance, document that LMX moderates the relationship between social undermining and employee silence. In socialization literature, there has been a lack of empirical evidence regarding the moderating role of LMX in the relationship between orientation training and work engagement. Based on the foregoing discussion, the fourth hypothesis of this study is therefore developed:

*Hypothesis 4*. LMX moderates the relationship between orientation training and work engagement in that this relationship is stronger when LMX is high than when it is low.

Education is an indicator of individual competence (Wang and Wang, 2016). In general, employees with higher education degrees typically possess more advanced cognitive, learning, information-seeking, and analytical decision-making skills than those with lower levels of education. For example, education is highly correlated with employees’ innovative behavior; and employees with higher education can better facilitate and engage in creative activities in the company (Kong, 2017). In the present study sample, hotel newcomers’ education level is likely to moderate the relationship between orientation training and PsyCap, meaning that those with higher education are more likely to make better use of organizational resources (e.g., effective orientation training) to foster their personal resources (e.g., PsyCap). In particular, higher education level frequently denotes more vital personal abilities (e.g., learning, cognition, etc.), which aids in enabling new hotel employees to learn and comprehend the pertinent training materials more quickly, effectively converting the resource advantage into a psychological advantage and having higher PsyCap. In this respect, most early studies (e.g., Kong, 2017) concentrate on the impact of education on corporate performance. In the domain of organizational socialization research, Liu et al. (2023) report that income moderates the relationship between task mastery and work engagement. An argument could be extended to expect that income moderates the relationship between orientation training and work engagement. Cai et al. (2021) and Harris et al. (2022) also call for more empirical investigations on the moderating effect in newcomers’ organizational socialization research. Therefore, the fifth hypothesis of this study is developed:

*Hypothesis 5*. The relationship between orientation training and PsyCap is moderated by newcomer education in that this relationship is stronger when education is high than when it is low.

## 3. Methodology

### 3.1. Measurement scales

The questionnaire contains four core constructs (orientation training, PsyCap, LMX, and work engagement) whose measurement items are detailed in Appendix A. The four constructs each are measured by using a well-established Likert scale. Orientation training was measured by using Zheng’s (2018) scale ranging from 1 (very poor) to five (very good). Respondents were requested to indicate the levels of training effectiveness on, for example, orientation to organizational culture and history. PsyCap was measured using a 6-point Likert scale (1 = strongly disagree, 6 = strongly agree) developed by Luthans et al. (2007). An example is, “I always feel confident when presenting in front of many colleagues,” etc. LMX was measured by Graen and Uhl-Bien (1995) using a 5-point Likert scale (1 = strongly disagree, 5 = strongly agree), such as “I am clear about whether my leader is satisfied with my performance,” etc. Work engagement was measured by Schaufeli et al. (2006) using a 7-point Likert scale (0 = never, 6 = always), such as “I feel motivated at work,” etc. Finally, the study collected newcomers’ demographic information.

It should be mentioned that we took Greenbaum et al.’s (2018) suggestion by measuring LMX from employees’ perspectives for two main reasons. One is that our study focuses on exploring the behaviors and attitudes of a newcomer, and therefore his or her perceptions of LMX are critical. The other is that previous scholars (e.g., Minsky, 2002) measured LMX for leaders and subordinates but they found perceptual differences between leaders and associates. The differences are mainly due to communication problems.

### 3.2. Data collection and participants

Prior to formal data collection, we conducted a pilot test and collected 20 copies of questionnaires from hotel newcomers. The Cronbach alpha value of each latent constructs in the questionnaires was greater than 0.90. In the main study, respondents must meet the following two preconditions: (a) non-managerial employees from 3- to 5-star hotels and (b) work tenures between 1 to 12 months following entry into the employment organization. We used snowball sampling technique to collect the empirical data. The formal data were collected between December 2021 and April 2022. We adopted the strategy of online data collection by using the “Wenjuanxing” platform^2^ for two notable reasons. One is that this platform is widely used in China (Wang et al., 2021). The other is that offline data collection was impossible due to the COVID-19 problem. We motivated respondents’ engagement to fill out the questionnaires by using a random bonus package ranging from 1–10 RMB yuan. As a result, 674 questionnaires were returned, among which 77 invalid questionnaires (e.g., responding time being less than 90 s) were removed, and 567 valid questionnaires were retained. The socio-demographic characteristics are presented in Table 1.

**Table 1:** Demographic characteristic.

Respondents characteristics	Frequency	Percentage (%)
*Gender*		
Male	197	34.7
Female	370	65.3
*Age*		
≤ 20	73	12.9
21–25	267	47.1
26–30	101	17.8
31–35	63	11.1
36–40	29	5.1
≥ 41	34	6.0
*Education*		
High school/junior college and below	183	32.3
University/College or above	384	67.7
*Organizational tenures*		
1–3 months	158	27.9
4–6 months	137	24.1
7–9 months	109	19.3
10–12 months	163	28.7
*Income*		
$315 and below	113	19.9
$316–630	302	53.3
$631–945	90	15.9
$946 and above	62	10.9
*Position*		
General Employees	453	79.8
Foreman / Supervisor 3-star hotel	114	20.1
*Hotel Star 4-star hotel 5-star hotel*		
3-star hotel	73	12.9
4-star hotel	234	41.3
5-star hotel	260	45.8

### 3.3. Common method variance/bias

To minimize the effect of common method variance/bias (CMV/B), Podsakoff et al. (2003) suggest the use of different scales for different constructs. Therefore, the points of the Likert scales among the four constructs in the present study differ from each other. Moreover, respondents’ anonymity was assured, and their engagement in filling out the questionnaire was motivated (noted earlier). All these strategies are effective and helpful in minimizing the effect of CMV/B. Apart from the foregoing, two more methods were adopted to detect the potential problem.

Specifically, one post-hoc test (i.e., one-factor method) was conducted to detect the CMV/B problem. All the measurement items for the four latent constructs were entered in SPSS 26.0, and principal component factor analysis was analyzed. The principal component factors turned out to be not a single common factor but multiple factors. The first factor explains 18.80% of the total variance, which is far below the threshold level of 50% (Harman, 1976).

The other post-ho test involves the unmeasured latent method construct (ULMC, Richardson et al., 2009) method. In particular, we have built four different models using AMOS 24.0: (a) model A (the trait-only model, *χ^2^/df* = 4.155), (b) model B (the method-only model, *χ^2^/df* = 9.853), (c) model C (the trait/method model, *χ^2^/df* = =3.655), and (d) model D (the trait/method -R model, *χ^2^/df* = 3.077). The results show that the trait-only model (i.e., model A) fits better (∆*χ^2^* = 4373.231, *p* = 0.000) than the method-only model (i.e., model B), suggesting that observed variance in the independent and dependent constructs is not because of method alone. The trait/method model (i.e., model C) fits better than (∆*χ^2^* = 227.700, *p* = 0.000) the trait-only model (i.e., model A), which shows that trait-based and method variance is presented in the data. Finally, the revised trait/method model (i.e., model D) fit better (∆*χ^2^* = 700.438, *p* = 0.000) than the trait/method model (i.e., model C), providing no substantial evidence of bias because of CMV.

## 4. Results

### 4.1. Reliability, validity and model Fit

The data were analyzed using SmartPLS 3.0. This method is suitable for complex models such as the one (with both moderation and mediation) in the present study. In addition, most of the causal paths in the present study are exploratory, and PLS-SEM is, as per Hair et al. (2017), a suitable method. Generally, the reliability and validity of the theoretical constructs have been guaranteed.

First, the factor loading of each construct’s specific items was tested, and their values were between 0.732 and 0.864 (see Table 2), higher than the threshold level of 0.7 (Hair et al., 2017). Second, the composite reliability (CR) ranges from 0.936–0.955 (see Table 2), which is greater than the criterion of 0.7, indicating that each latent construct exhibits a high level of internal consistency (Hair et al., 2017).

**Table 2:** Assessment results of the overall measurement model.

Constructs	Items	Loadings	*T* Statistics	Cronbachα’s α	CR	AVE
Orientation training	OT1	0.770	35.229	0.936	0.945	0.635
	OT2	0.734	30.004			
	OT3	0.732	31.093			
	OT4	0.805	44.284			
	OT5	0.799	39.974			
	OT6	0.832	53.694			
	OT7	0.829	52.529			
	OT8	0.825	47.717			
	OT9	0.767	33.110			
	OT10	0.864	69.789			
PsyCap	PC1	0.788	39.410	0.948	0.955	0.639
	PC2	0.814	50.360			
	PC3	0.794	47.660			
	PC4	0.833	54.552			
	PC5	0.754	34.680			
	PC6	0.837	48.322			
	PC7	0.835	47.762			
	PC8	0.774	37.172			
	PC9	0.783	26.532			
	PC10	0.750	23.030			
	PC11	0.843	60.103			
	PC12	0.775	27.222			
Work engagement	WE1	0.827	44.312	0.945	0.953	0.671
	WE2	0.858	56.503			
	WE3	0.832	46.535			
	WE4	0.788	33.333			
	WE5	0.809	32.541			
	WE6	0.788	38.562			
	WE7	0.855	60.274			
	WE8	0.840	50.051			
	WE9	0.798	32.540			
	WE10	0.791	34.337			
LMX	LMX1	0.799	43.131	0.919	0.936	0.675
	LMX2	0.844	55.527			
	LMX3	0.841	48.224			
	LMX4	0.816	45.303			
	LMX5	0.825	51.814			
	LMX6	0.786	38.913			
	LMX7	0.838	55.998			

(1) 567 parent samples with 5,000 bootstraps. (2) SmartPLS 3.0 software was used to analyze the data. (3) Factor loading values are all statistically significant at 0.001 level. (4) SRMR: Saturate model = 0.029, Estimate model = 0.032. (5) Control variables include age, gender, income, education, and position, among which education is moderator as well. (6)VIF values range from 1.899 to 3.700.

In terms of convergent validity, Hair et al. (2017) suggest that the AVE value of a construct should be greater than 0.50. The present study’s AVE values range from 0.635 to 0.675 (see Table 2), all greater than the foregoing threshold level. With regard to discriminant validity, we used the following two approaches to assess it. The first one concerns the square root of each AVE value. Table 3 indicates that the square root of each AVE value on the diagonal is greater than the correlation coefficients between the two corresponding constructs. This would suggest that all four variables in our study have good discriminant validity according to the criteria suggested by Ab Hamid et al. (2017) and Hair et al. (2017). The second discriminant validity assessment involves, as per Henseler et al. (2015), the HTMT (Heterotrait-Monotrait) Ratio test. In particular, when HTMT value is less than 0.85, the corresponding construct can be considered to have good discriminant validity (Henseler et al., 2015). In the present study, HTMT values in Table 4 show that all the four latent constructs exhibit acceptable level of discriminant validity.

**Table 3:** Constructs’ correlations and squared root values of AVE.

Constructs	LMX	OT	WE	PsyCap
LMX	**0.822**			
Orientation training(OT)	0.593	**0.797**		
Work engagement(WE)	0.643	0.456	**0.819**	
PsyCap	0.718	0.629	0.682	**0.799**

(1) *N* = 567. (2) Square root values of AVE are shown on the diagonal. (3) Correlation values are below the diagonal and statistically significant at 0.001 level. (4) Values were obtained by using SmartPLS 3.0 software.

**Table 4:** HTMT discriminant validity of the constructs.

Constructs	LMX	OT	WE	PsyCap
LMX	NA			
Orientation training(OT)	0.637	NA		
Work engagement(WE)	0.687	0.480	NA	
PsyCap	0.766	0.662	0.717	NA

HTMT, heterotrait–monotrait Ratio. NA, not applicable. HTMT ratios are below the diagonal.

Turning to the model fit, Hair et al. (2017) suggest that the SRMR (Standardized Root Mean Residual) value should be less than 0.08. In the present study, SRMR values in the Saturated and Estimated models are 0.029 and 0.032, respectively. These results suggest that the model fits the data well in the present study. Finally, Hair et al. (2017) suggest that VIF values of measurement items should be less than 5.00. In the present study, VIF values range from 1.889 to 3.700. This would suggest that VIF problem is not an issue in our study.

### 4.2. Hypothesis testing results

We tested all hypotheses in this study using 567 parent samples with 5,000 complete bootstraps enabled in SmartPLS 3.0. Specifically, we chose bias-corrected complete bootstrapping to test research hypotheses at the significance level of 0.05 (2-tailed). One advantage of this method, as per Hair et al. (2019), lies in its ability in working around the potential estimation bias caused by multivariate nonnormal data. As a result, our hypothesis testing results are less likely to be biased as per Hair et al. (2019) and the results are summarized in Table 5. In particular, H1 concerns the effect of orientation training on PsyCap, and it gains empirical support (*β* = 0.608, *p* = 0.000) in this study. H2 involves the effect of PsyCap on newcomers’ work engagement, and it has also been supported (*β* = 0.432, *p* = 0.000) in the present empirical data. H3 relates to the mediating role of PsyCap, which has proven it to be a substantial (*β* = 0.257, *p* = 0.000) mediator between orientation training and work engagement. Moreover, PsyCap’s mediating role has been further confirmed by the following situations. As shown in Table 5, when controlling for PsyCap as a mediator, path C was not significant (*β* = −0.020, *p* = 0.638); but when PsyCap is not being controlled in the model with only one antecedent (i.e., orientation training) and one outcome (work engagement), path C′ was, however, significant (*β* = 0.432, *p* = 0.000).

**Table 5:** Summary of direct and indirect effects.

Path	Coefficients	BC-CIs [2.5%; 97.5%]	*T* Statistics	*p*-values
*Direct effects*				
OT → PsyCap(H1)	0.608	[0.518; 0.673]	15.877	0.000^**^
PsyCap → WE(H2)	0.432	[0.337; 0.522]	9.054	0.000^**^
OT → WE(C)	−0.020	[−0.101; 0.064]	0.471	0.638
OT → WE(C′)	0.432	[0.352; 0.510]	10.725	0.000^**^
*Mediation effects*				
OT → PsyCap → WE(H3)	0.263	[0.201; 0.331]	7.886	0.000^**^
*Moderation effects*				
OT*LMX → WE(H4)	0.065	[0.011; 0.124]	2.238	0.025^*^
OT*Education → PsyCap(H5)	0.096	[0.006 0.173]	2.241	0.027^*^

The 567 samples were Bootstrapped 5,000 times by SmartPLS 3.0, and the test type was a two-tailed test with significance level *p* = 0.05; ^*^ indicates *p* < 0.05, ^**^ indicates *p* < 0.01 (two-tailed).

**Figure 1:**
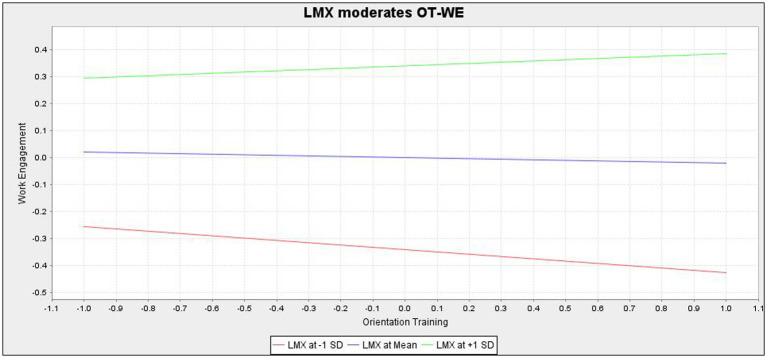
LMX moderates the relationship between orientation training and work engagement.

H4 and H5 are moderation hypotheses. Specifically, H4 hypothesizes that LMX moderates the relationship between orientation training (independent variable) and work engagement (dependent variable). Results in Table 5 indicate that the interaction variable (i.e., LMX*orientation training) significantly (*β* = 0.065, *p* = 0.025) influences work engagement. The moderation plot is shown in Figure 1. A post-hoc group regression analysis was conducted using PLS-SEM3.0, and the results show that in terms of the positive effect of orientation training on engagement, new employees with high LMX have been more affected by engagement (*β* = 0.236, *p* = 0.000) than those with low LMX (*β* = 0.219, *p* = 0.000). In other words, results reveal that newcomers with high LMX were more effective in using social resources such as organizational orientation training to increase their engagement than their counterparts with low LMX. Jointly, the foregoing results would suggest that H4 gains empirical support in this study.

**Figure 2:**
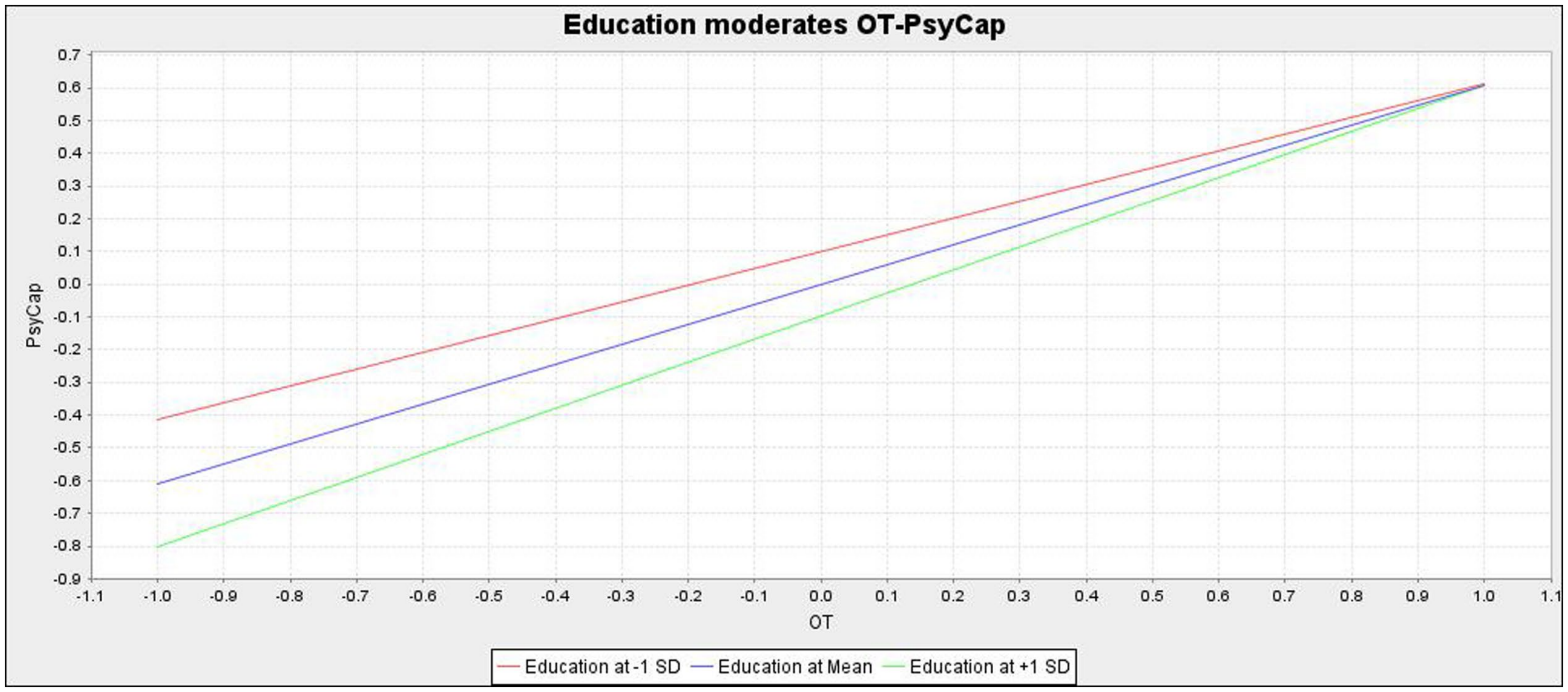
Education moderates the relationship between orientation training on PsyCap.

Likewise, H5 hypothesizes that education plays a moderating role between orientation training (independent variable) and PsyCap (dependent variable). Table 5 shows that the variable (i.e., education*orientation training) significantly (*β* = 0.096, *p* = 0.027) affects PsyCap. The moderation plot is shown in Figure 2. Furthermore, a post-hoc group regression analysis was conducted using PLS-SEM3.0. The results show that in terms of the positive effect of orientation training on PsyCap, new employees with education at college and above were more affected by PsyCap (*β* = 0.674, *p* = 0.000) than those with education at high school and below (*β* = 0.560, *p* = 0.000). In other words, newcomers with higher education (college and above) are more likely to make good use of socialization resources (i.e., orientation training) to maintain and develop their PsyCap levels than their counterpart group of lower education (high school and below). Taken together with the foregoing results, H5 is empirically supported in the present data.

## 5. Discussion

### 5.1. Originalities and contributions

In the domain of organizational socialization, the research framework in this study is one of the few studies (e.g., Liu et al., 2023) that are theoretically and jointly built on COR theory and SRT theory. In general, our research framework as well as its study findings helps managers and the academic community understand how, why, and under what conditions orientation training affects newcomers’ work engagement. Our empirical findings add to the body of knowledge on organizational socialization and broaden and deepen the explanatory capacity of COR theory and SRT theory on the phenomena of newcomers’ organizational integration in several ways.

First, there has been a lack of documented report on the direct and positive effect of orientation training on newcomers’ PsyCap; and our study provides the empirical evidence on this research hypothesis (H1). This would suggest that Saks and Gruman’s (2018) theoretical notion of this particular causal linkage gains empirical support successfully. This also lends empirical support for the argument of COR: organizational resources have spillover effect on personal resources (Hobfoll et al., 2018). The finding regarding the direct linkage between orientation training and PsyCap is empirically exploratory and thus contributes to the literature substantially. Apart from orientation training, future studies should both empirically investigate more organizational and job resources as listed in Saks and Gruman’s (2011) work and examine their impact on newcomers’ PsyCap. Future studies are particularly warranted to examine other types of orientations—e.g., Wen et al.’s (2023) cultural orientation—as well as their influences on socialization process and outcomes.

Second, COR theory proposes that resources do not exist individually and separately, but usually present themselves in a cluster (Hobfoll et al., 2018). In line with this proposition, the four newcomers’ four HERO resources should be considered and examined as a cluster of PsyCap. However, more often than not, most previous socialization research examines HERO as four independent resources only (e.g., Self-efficacy, Bauer et al., 2007), with a neglect of treating HERO as a cluster of PsyCap with only a few exceptions (e.g., Ngo et al., 2023). The empirical findings regarding PsyCap as well as its relationship with work engagement, have been accordingly very rare. For instance, Karatepe and Karadas (2015) investigate only self-efficacy as well as its effect on work engagement. The present study differs from most previous socialization research. That is, we theoretically consider PsyCap, as per Luthans and Youssef-Morgan (2017), as a theoretical construct which is organically built on its four HERO components. In this study, PsyCap has proven itself to be a theoretical construct with both convergent and discriminant validity. As such, we lend empirical support for both COR (Hobfoll et al., 2018) and PsyCap (Luthans and Youssef-Morgan, 2017) theories in that PsyCap is a resource caravan rather than four independent HERO resources. This drops an important theoretical implication that future research should continue to consider PsyCap as a distinct and legitimate core construct and take a step further to examine more nomological network of PsyCap (e.g., employee development policies, Ngo et al., 2023) for one main reason. That is, SRT theory emphasizes that the four individual HERO resources “operate synergistically, and their overall PsyCap may demonstrate the most vital relationship with socialization outcomes (Saks and Gruman, 2011, p. 6).”

Third, in their SRT model of engagement pathway to organizational socialization, Saks and Gruman (2018) propose that socialization resources (e.g., orientation training) have both direct and indirect effects on work engagement. This proposition has only been partially supported in the present study. Specifically, Table 5 indicates that orientation training predicts work engagement directly without controlling for the mediator of PsyCap (*β* = 0.432, *p* = 0.000). After controlling for the mediator, the direct effect of orientation training on work engagement disappears (*β* = −0.020, *p* = 0.683). This reveals that PsyCap is a full, rather than partial, mediator in the relationship between orientation training and work engagement. This suggests that SRT model of engagement pathway to socialization (Saks and Gruman, 2018) needs to be refined when socialization resources and personal resources are narrowed down to the specific resources of orientation training and PsyCap. The refinement concerns the foregoing direct versus indirect effect of orientation training on work engagement. It is particularly felt considering that SRT approach to socialization research is a new paradigm, and SRT is still in its infancy (Ashforth et al., 2012; Cai et al., 2021).

**Figure 3:**
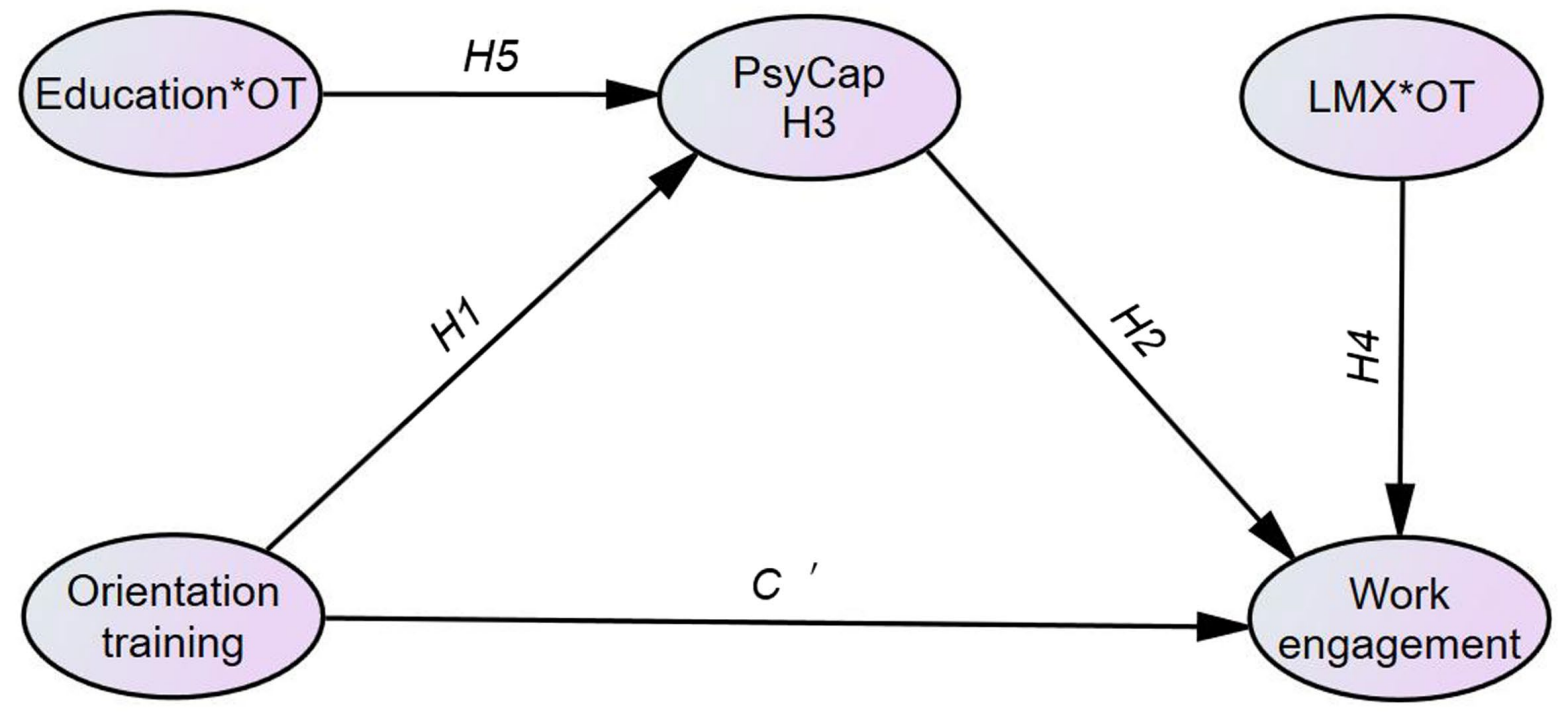
Conceptual framework of this study. 1. OT=Orientation training, WE=Work engagement, LMX=Leader-member exchange. 2. Path C concerns the direct effect of OT on WE without controlling for the PsyCap mediators; after controlling for the mediators, path C is accordingly changed into path C'. 3. Control variables include gender, age, position, and income. 4. * indicates that LMX and Education are moderators.

Finally, in response to the relative lack of moderating effects in organizational socialization (Cai et al., 2021), we have examined the moderating roles of LMX and educational level in the research framework (Figure 3). In particular, we consider LMX as a resource caravan passageway in the process of organizational socialization, and its quality is likely to regulate the speed of various resource fleets on the “socialization path” and its impact on work engagement. Our empirical result indicates that the effect of orientation training on work engagement is stronger when LMX is high than when it is low. This finding is exploratory and thus significantly contributes to the body of literature. This provides empirical support for one notion of COR (Hobfoll et al., 2018): “LMX is a resource passageway, and it plays an important role in follower engagement” (p. 9).

In terms of the positive effect of orientation training on PsyCap, our empirical result reveals that this effect is stronger when education is high than when it is low. Education is a type of energy resource, as opposed to LMX, which makes up the general environment of resource passageway (Hobfoll et al., 2018). The finding regarding the moderation role of education in the present study is essentially exploratory. Therefore, it is suggested that energy resources, as well as their roles in the socialization process, should be examined and included in future SRT models.

### 5.2. Practical implications

In the present study, the effect of orientation training on work engagement is fully mediated by PsyCap. In other words, organizational resources (e.g., orientation training) affect personal resources (e.g., PsyCap), which in turn affect work engagement. This would suggest that orientation training must be ensured to be positive and effective. Otherwise, ineffective training leads to lower level of PsyCap, which in turn results in disengagement. As such, orientation training programs should be well designed, implemented, and evaluated from time to time so that both trainers and trainees are well engaged in achieving the effectiveness of orientation training.

In practice, hotel training is oriented, more often than not, to newcomers’ skills and abilities but not to their psychological state, such as PsyCap. In the present study, PsyCap has proven itself as a resource caravan and it influences work engagement positively. One practical implication is that following their entry into the organization, newcomers should be tested for their PsyCap levels from time to time. This is due to PsyCap’s nature of plasticity, malleability, and openness to change (Luthans and Youssef-Morgan, 2017). In case of new hires with low PsyCap levels, managers should diagnose and intervene in such problems. The major objective of PsyCap development programs would not necessarily be building new knowledge and skills, but intervening and enhancing, for instance, newcomers HERO in that they can do well by means of using existing and potential resources. Special programs like positive experiences, performance feedback, and effective coaching/modeling could be developed and tailored to augment newcomers’ PsyCap levels. In short, PsyCap is subject to change, and once higher levels of newcomers’ PsyCap have been developed and maintained, higher levels of work engagement will be accordingly developed.

The present study has examined two moderators including LMX and education. In particular, newcomers with college and above education make better use of training resources than their counterparts with senior middle school and below education to cultivate PsyCap. This leads to the following implication—It is important to recruit employees, particularly the front-line staff, with an education background of college or above. The other moderator is LMX, a resource caravan passageway (Liu et al., 2023). LMX is a way for managers and subordinates to achieve resource flow. High-quality LMX can provide newcomers with various explicit resources (e.g., information bias) and implicit resources (e.g., supervisor support). Low-quality LMX means managers and subordinates are often filled with dissatisfaction, suspicion, and even resistance (Graen and Uhl-Bien, 1995). The results of our study suggest that LMX moderates the relationship between orientation training and newcomer engagement. This finding is exploratory in organizational socialization research domain. But it also echoes Xu et al. (2022) finding regarding the moderating role of LMX. Namely, in organizational context the interactive effect between LMX and social undermining affects employee silence significantly. As such, we extend the literature substantially. In the present study, the relationship between orientation training and PsyCap is specifically stronger when education is high than when it is low. Therefore, it is necessary for organizations to promote resource exchanges between managers and newcomers. The hotel can also set up a comparable prevention and intervention system so that when newcomers report low levels of LMX, hoteliers should diagnose the problem by conducting in-depth interviews, for example, to identify the root causes and quickly implement corresponding remedies.

### 5.3. Limitations and future studies

Our empirical data are self-reported and cross-sectional, which may lead to common method variance (CMV/B). These data collection methods have their limitations but are reasonable for two main reasons. First, Podsakoff et al. (2003) argue considerably on decreased or increased causal relationships. However, Lance et al. (2010) document that the common method’s inflationary bias is mitigated mainly by attenuation from measurement inaccuracy. Bauer et al. (2007) consider cross-sectional data a reasonable alternative strategy to capture the dynamics of newcomers’ organizational socialization phenomenon. To address CMV/B issue, we, therefore, conduct post-hoc statistical analysis to diagnose the potential CMV/B problem. As noted earlier (Section 3.3 Common Method Variance/Bias), the statistical results indicate no substantial evidence of bias because of CMV. Furthermore, to lessen method bias, techniques such as ensuring respondent anonymity, as advised by Podsakoff et al. (2003), were utilized at the response reporting stage. Second, all the latent constructs in our overall models are generated from psychological and subjective impressions that are intangible but perceptible. As a result, the self-reporting approach is suitable, as per Song and Chathoth (2011), in that people’s true perceptions and intents can typically be reported by no one else but themselves.

Because most of our study findings are exploratory, the generalizability of our study findings is unknown. Given this limitation, it is necessary to verify our study findings in future studies with longitudinal data and in other service industries, national cultures, and geographical areas. Future studies should investigate the roles of more socialization resources (e.g., organizational support) as listed in SRT (Saks and Gruman, 2018) and personal resources (e.g., core self-evaluations) in the development of newcomers’ work engagement. Finally, more moderators (e.g., emotional intelligence) should be investigated in future SRT models.

## 6. Concluding remarks

Built on COR and SRT theories, this study has developed and validated an integrative model of work engagement. The empirical results reveal that PsyCap fully mediates the relationships between orientation training and work engagement. They also show that education and LMX each moderates the effects of orientation training on PsyCap and work engagement, respectively. These findings are empirically exploratory, insightful, and valuable, contributing to the literature substantially. These originalities have particularly felt in consideration of the following two facts: (a) the engagement pathway of SRT to studying newcomer socialization has been a new paradigm and trend, and (b) there has been only a small number of empirical evidence on SRT theoretical propositions. Future work engagement pathway to studying newcomer organizational socialization should include more constructs of socialization resources, personal resource caravan, and resource passageways. Future studies are also warranted to conduct a comparative study in a Western context, other service industries, and other countries/regions. Finally, in terms of why and under what conditions orientation training affects newcomers’ work engagement, this research provides, nevertheless, both practical and theoretical contributions for future research scholars to build on.

## Data availability statement

The original contributions presented in the study are included in the article/Supplementary material, further inquiries can be directed to the corresponding author.

## Ethics statement

Ethical review and approval was not required for the study on human participants in accordance with the local legislation and institutional requirements. Written informed consent from the [patients/ participants OR patients/participants legal guardian/next of kin] was not required to participate in this study in accordance with the national legislation and the institutional requirements.

## Author contributions

ZS: Formal analysis, Resources, Writing – original draft, Conceptualization, Funding acquisition, Investigation, Methodology, Software, Supervision, Writing – review & editing. HZ: Formal analysis, Resources, Writing – original draft, Data curation, Project administration. JL: Data curation, Resources, Supervision, Validation, Visualization, Writing – review & editing.

## Funding

The author(s) declare financial support was received for the research, authorship, and/or publication of this article. This work was supported by the National Natural Science Fund of PR China under Grant 72262011.

## Conflict of interest

The authors declare that the research was conducted in the absence of any commercial or financial relationships that could be construed as a potential conflict of interest.

## Publisher’s note

All claims expressed in this article are solely those of the authors and do not necessarily represent those of their affiliated organizations, or those of the publisher, the editors and the reviewers. Any product that may be evaluated in this article, or claim that may be made by its manufacturer, is not guaranteed or endorsed by the publisher.

## Footnotes

^1^State of the Global Workplace Report. 2021. Available online: https://www.gallup.com/workplace/349484/state-of-theglobal-workplace.aspx? (accessed on May 6, 2023).

^2^ https://www.wjx.cn
