# Supplementary material for: Exploring the mediating role of PsyCap in the relationship between orientation training and work engagement: the perspective of COR and SRT theories
Source: Front Psychol. 2023 Sep 25;14:1263658. doi: 10.3389/fpsyg.2023.1263658 (PMC10561258; doi:10.3389/fpsyg.2023.1263658)
Supplement: Supplementary file 1 [file Data_Sheet_1.PDF]

**Appendix A.** Measurement items used in present study.

**Orientation training (OT)**

OT1. Effectiveness of the hotel in providing training on the organization's profile (e.g., about the history and culture of our hotel and the current state of development, etc.).

OT2. Effectiveness of the hotel in providing training on safety and fire prevention knowledge.

OT3. The hotel offers other training (such as English and computers, etc.) in effect.

OT4. The effectiveness of teacher-centered training methods and approaches (e.g., teacher speaks, student listens, etc.) in hotel orientation training.

OT5. Effectiveness of participant-centered training methods and approaches (e.g., participant visits, practical exercises, etc.) in hotel orientation training.

OT6. The effectiveness of the interactive training (e.g. Q&A, case studies, scenarios, etc.) adopted by the hotel.

OT7. Effectiveness of departmental information-type training (e.g., departmental overview, product knowledge, notes, etc.) provided by the department.

OT8. Effectiveness of training provided by the department on job skills categories (e.g. job skills, standard operations, system software, etc.).

OT9. The effect of the department using formal guidance (e.g., career development planning communication, etc.).

OT10. The actual effectiveness of departmental orientation training compared to your own expectations.

**PsyCap**

PC1. I feel confident in representing my work area in meetings with management.

PC2. I feel confident contributing to discussions about the company’s strategy.

PC3. I feel confident presenting information to a group of colleagues.

PC4. If I should find myself in a jam at work, I could think of many ways to get out of it.

PC5. Right now, I see myself as being pretty successful at work.

PC6. I can think of many ways to reach my current work goals.

PC7. At this time, I am meeting the work goals that I have set for myself.

PC8. I can be “on my own,” so to speak, at work if I have to.

PC9. I usually take stressful things at work in stride.

PC10. I can get through difficult times at work because I’ve experienced difficulty before.

PC11. I always look on the bright side of things regarding my job.

PC12. I’m optimistic about what will happen to me in the future as it pertains to work.

**Work engagement (WE)**

WE1. At my work, I feel bursting with energy.

WE2. At my job, I feel strong and vigorous.

WE3. When I get up in the morning, I feel like going to work.

WE4. I can continue working for very long periods at a time.

WE5. At my work, I always persevere, even when things do not go well.

WE6. To me, my job is challenging.

WE7. I feel happy when I am working intensely.

WE8. I get carried away when I am working.

WE9. When I am working, I forget everything else around me.

WE10. It is difficult to detach myself from my job.

**Leader–member exchange (LMX)**

LMX1. I usually know whether or not my leader is satisfied with what I do.

LMX2. My leader understands my job problems and needs very well.

LMX3. My leader recognizes my potential very well.

LMX4. At the workplace, my leader would help me to solve difficult problems.

LMX5. At the workplace, my leader would “bail me out” at their expense.

LMX6. I have enough confidence in my leader that I would defend and justify their decision if they were not present to do so.

LMX7. I have very good relationships with my leader.
